# Late tamoxifen in patients previously operated for breast cancer without postoperative tamoxifen: 5-year results of a single institution randomised study

**DOI:** 10.1186/1471-2407-10-205

**Published:** 2010-05-14

**Authors:** Andrea Veronesi, GianMaria Miolo, Maria D Magri, Diana Crivellari, Simona Scalone, Ettore Bidoli, Davide Lombardi

**Affiliations:** 1 Division of Medical Oncology C, National Cancer Institute, Via Franco Gallini 2, 33081 Aviano, Italy; 2 Epidemiology Unit, National Cancer Institute, Via Franco Gallini 2, 33081 Aviano, Italy

## Abstract

**Background:**

A population of breast cancer patients exists who, for various reasons, never received adjuvant post-operative tamoxifen (TAM). This study was aimed to evaluate the role of late TAM in these patients.

**Methods:**

From 1997 to 2003, patients aged 35 to 75 years, operated more than 2 years previously for monolateral breast cancer without adjuvant TAM, with no signs of metastases and no contraindication to TAM were randomized to TAM 20 mg/day orally for 2 years or follow-up alone. Events were categorized as locoregional relapse, distant metastases, metachronous breast cancer, tumours other than breast cancer and death from any causes, whichever occurred first. The sample size (197 patients per arm, plus 10% allowance) was based on the assumption of a 30% decrease in the number of events occurring at a rate of 5% annually in the 10 years following randomization. Four hundred and thirty-three patients were randomized in the study (TAM 217, follow-up 216). Patients characteristics (TAM/follow-up) included: median age 55/55 years, median time from surgery 25/25 months (range, 25-288/25-294), in situ carcinoma 18/24, oestrogen receptor (ER) positive in 75/68, negative in 70/57, unknown in 72/91 patients. Previous adjuvant treatment included chemotherapy in 131/120 and an LHRH analogue in 11/13 patients.

**Results:**

Thirty-six patients prematurely discontinued TAM after a median of 1 month, mostly because of subjective intolerance. Eighty-three events (TAM 39, follow-up 44) occurred: locoregional relapse in 10/8, distant metastases in 14/16, metachronous breast cancer in 4/10, other tumours in 11/10 patients. Less ER-positive secondary breast cancers occurred in the TAM treated patients than in follow-up patients (1 vs 10, p = 0.005). Event-free survival was similar in both groups of patients.

**Conclusions:**

This 5-year analysis revealed significantly less metachronous ER-positive breast cancers in the TAM treated patients. No other statistically significant differences have emerged thus far.

## Background

Breast cancer patients present a lifelong increased risk of a contralateral new breast cancer, with a reported incidence of 0.5-1% annually, translating into a 10-20% risk in long-term survivors [1-4]. In addition, local relapses and distant metastases occur even long time after local curative treatment [5].

Tamoxifen (TAM) has a well defined role in the postoperative management of oestrogen receptor (ER) positive breast cancer with a significant impact upon locoregional relapse, the development of distant metastases and of contralateral metachronous breast cancer [5]. In addition, randomised studies have shown that TAM is able to reduce the incidence of primary breast cancer in various settings: (high-risk patients [6-8] and hysterectomised low-risk patients [9]).

In spite of the widespread use of TAM, a large population of breast cancer patients who never received TAM exists, mostly going back to past decades, either because of the low risk of relapse or because only chemotherapy was planned.

Whether this population of breast cancer patients still has a chance to derive the well known benefits of TAM is not known.

The present randomised study was aimed to evaluate the role of late TAM in patients previously operated for breast cancer who did not receive postoperative TAM.

The 5-year results of this study are the subject of the present report.

## Methods

From July 1997 to May 2003 all eligible patients seen at our Institution were considered for the study. Eligibility criteria included histologically proven infiltrating or in situ breast cancer, age between 35 and 75 years, radical surgery for monolateral breast cancer more than 2 years previously, no signs of breast cancer, no contraindication to TAM and informed consent.

Disease stage at the time of surgery was retrospectively classified according to the TNM classification. ER status was defined as positive when ≥10% of the tumour cells expressed ER by immunohistochemical assay or when >10 fmol/mg of cytosol protein by ligand-binding assay were present.

A recent negative mammogram, chest X-rays, bone scan and liver ultrasound were requested before randomization.

Patients were stratified according to the time elapsed from local treatment (2-5 years versus more than 5 years) and randomised to TAM 20 mg/day orally for 2 years or follow-up alone.

The follow-up procedures were identical in the two arms and included patient history, a physical examination and serum biochemistry with Ca 15.3 every 6 months for the first 2 years and yearly thereafter, an annual gynaecologic evaluation and an annual mammogram. All other examinations were planned only if symptoms occurred.

### Statistical considerations

The primary endpoint was event-free survival. Events were categorised as locoregional relapse, distant metastases, metachronous breast cancer, secondary tumours other than breast cancer and death from any other causes, whichever occurred first. Event-free survival was defined as the time between randomisation and the manifestation of an event.

Secondary endpoints included overall survival and the toxicity profile. The toxic effects of TAM were categorized according to the WHO criteria [10]. Overall survival was defined as the time interval between randomisation and death for any cause.

Comparison of proportions was done by means of Chi-square analysis of Fisher's exact test when appropriate. The Kaplan Meier method [11] was used to plot event-free survival and overall survival. The statistical analyses were carried out using the SAS Software version 9.13 (SAS Institute Inc., Cary, North Carolina, USA).

The sample size (197 patients per arm, plus 10% allowance) was based on the assumption of a 30% decrease in the number of events occurring at a rate of 5% yearly in the 10 years following randomization.

The study was approved by the institutional Ethical Committee (registration number: CRO-14-1997).

## Results

From March 1997 to May 2003, 433 patients were randomized in the study (TAM 217, follow-up 216). The main characteristics of the patients are reported in Table 1.

**Table 1:** Patient Characteristics

	TAM (n = 217)	FU (n = 216)	p value
**Median age (range)**	**55 yrs (28-75)**	**55 yrs (26-75)**	

Time from surgery			
2-5 years	121	120	p = 0.97
>5 years	96	96	

Median time from surgery in months (range)	25 (25-288)	25 (25-294)	p = 0.99

Menopausal status			
Premenopausal	33	33	p = 0.98
Postmenopausal	184	183	

Stage at diagnosis			
Tis	18	24	
I	84	82	
II	87	88	p = 0.52
III	28	18	
Unknown	2	3	

Nodal status			
Positive	74	76	
Negative	126	119	p = 0.72
Unknown	17	21	

Previous hysterectomy	39	36	p = 0.72

Er status			
ER+	75	68	
ER-	68	58	p = 0.21
ER unknown	73	91	

Previous medical treatment			
Anthracyclines	44	40	
CMF	87	80	
LH-RH analogue for 2 years	11	13	p = 0.79
No treatment	75	83	

**Figure 1:**
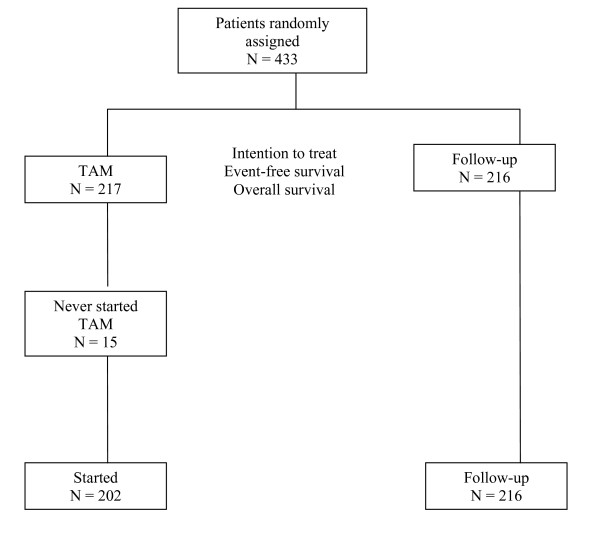
**Consort Diagram**.

Figure 1 represents a CONSORT diagram for the study.

Of the 217 patients randomized to the TAM group, 15 never started the therapy.

In general, TAM was well tolerated and no G4 events were recorded. There were no statistically significant differences in the side effects between the two arms, apart from hot flashes which occurred more frequently in the TAM patients (51%) than in those in follow-up (5%).

Thirty-six patients discontinued TAM after a median of one month due to toxic effects or subjective intolerance. The reasons for discontinuation are reported in Table 2.

**Table 2:** Reasons for Tam Discontinuation

Subjective intolerance	7
Phlebitis/vascular effects	5

Skin rash	4

Allergy	2

Anxiety	2

Cardiopalm/arrhythmia	2

Insomnia	2

Vertigo	2

Headache	2

Vaginitis	4

Other	4

At the time of analysis, the median follow-up period for the TAM group was 89 months, while for the follow-up group this was 88 months.

The number and type of events are summarized in Table 3.

**Table 3:** Events

	TAM (n = 217)	FU (n = 216)	p value
N. of events	39	44	p = 0.53

Local relapse	10	8	p = 0.64

Contralateral BC	4	10	p = 0.11
ER+	1	10	p = 0.005
ER-	3	0	p = 0.24

Distant metastases - Lung - Liver - Brain - Bone - Lymph nodes - Peritoneal carcinosis	14 7 0 3 2 1 1	16 2 3 1 6 4 0	p = 0.70 p = 0.18 p = 0.12 p = 0.62 p = 0.18 p = 0.22 p = 1.00

Second primary neoplasm	11	10	p = 1.00

Endometrial cancer	1	0	p = 1.00

Thirty-nine events occurred in the TAM group while 44 events occurred in the control group. The difference was not statistically significant.

Less ER-positive secondary breast cancers occurred in the TAM treated patients (p = 0.005). None of the other differences observed was statistically significant.

In the TAM group, 1 contralateral breast cancer occurred in the 75 patients whose original tumour was ER-positive, 1 in the 68 patients with ER-negative tumour and 2 in the 73 patients whose ER status was unknown. In the follow-up group, the corresponding figures were 1/68, 3/58 and 6/91. None of these differences were statistically significant.

**Figure 2:**
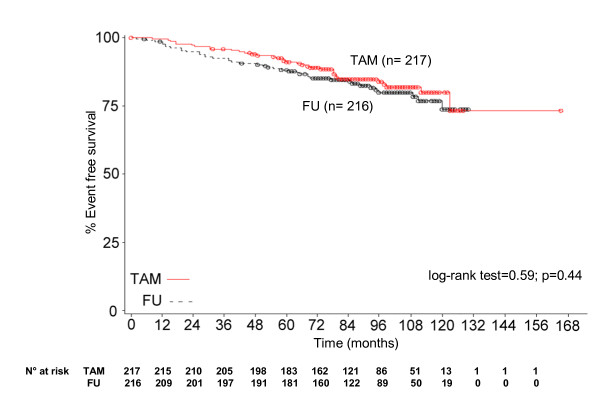
**Event-Free Survival (All randomized patients)**.

Event-free survival curves (all randomised patients) are reported in Figure 2. No statistically significant difference between the curves can be noted. The five-year event-free survival was 91% for the TAM patients and 88% for the follow-up group.

**Figure 3:**
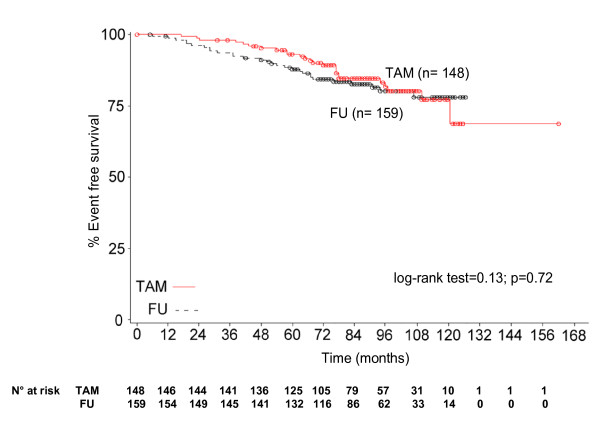
**Event-Free Survival (ER-positive patients)**.

Event-free survival curves of patients with known ER positivity are shown in Figure 3. Although there appears to be a trend towards a better event-free survival in the TAM treated group, the curves do not differ in a statistically significant manner. A planned subgroup analysis was performed according to the time from surgery (2-5 years vs more than 5 years) and adjuvant medical treatment (yes vs no). No statistically significant differences emerged (curves not shown).

Nineteen TAM patients and 18 follow-up patients died after a median of 67 and 53 months respectively. Survival curves (not shown) were superimposable.

## Discussion

TAM has proven to benefit all groups of patients with breast cancer and hormone sensitive tumours in the adjuvant setting and has a role in the prevention of breast cancer in several situations. The present study was aimed to investigate the role of TAM in patients operated for breast cancer who had never received TAM and it was powered to detect an effect in the order of that ascertained in the adjuvant setting.

The eligible population consisted partly of premenopausal patients who, several years before, did not receive TAM either because of a low risk of relapse or because they had received oophorectomy, chemotherapy or both. It should be noted that the acceptance of TAM as a standard therapy for premenopausal patients with hormone sensitive tumour was slower in Italy than in other countries and that, as a consequence, a certain population of patients diagnosed and treated in the Eighties and early Nineties who never received TAM according to the present indications exists. In addition, both pre- and postmenopausal patients who did not receive TAM either because their tumour was hormone insensitive or for other reasons, were eligible for the study. One hundred and sixty-four patients had an unknown ER status. Efforts were made to retrieve the information whenever possible, but some data of patients who had been operated elsewhere a long time before are missing. This may reflect the situation in contexts where it is less likely that the patients received tamoxifen as appropriate after primary treatment. Obviously, the lack of ER evaluation in a relevant proportion of the study population represents a major limitation of the study.

At this 5-year analysis, few events (n = 83) occurred and only a minority of these (n = 30) were of metastatic nature. This was predictable, taking into account the nature of the study in which the selection mechanism tended to exclude from randomization biologically aggressive tumours with a high propensity to early relapse. In addition, the majority of patients had received active adjuvant treatment, which may have contributed to the low number of events making difficult to ascertain the role of tamoxifen.

Some differences in outcome were detected in the two groups.

A statistically significant smaller number (1 vs 10) of ER-positive contralateral breast cancers, a trend towards fewer contralateral breast cancers (4 vs 10) and to a longer event-free survival in ER-positive cases were noted in the TAM-treated group. Contralateral ER-negative breast cancer occurred more frequently in the TAM group, supporting the inability of TAM to prevent ER-negative secondary tumours as previously described [12].

TAM was basically well tolerated and no serious adverse events occurred. The toxicity encountered was mostly related to the hormonal effects of TAM. Only one case of endometrial cancer occurred in a TAM-treated patient.

In the only published study with a design and size comparable to that of the present study, Delozier et al [13] noted 109 events in a population of 494 randomized patients followed-up for 10 years. An 83% 10-year disease-free survival in TAM- treated patients was reported, as compared to 75% in controls (p = 0.01). No difference in overall survival in the whole population was noted, but subgroups with node-positive or ER-positive disease had a better survival with TAM. Different from our study, TAM was planned to be administered continuously lifelong at the dose of 30 mg/day.

In a smaller study [14], 2 years of TAM had no influence upon disease-free survival, although there were more deaths (mostly unrelated to breast cancer) in the placebo group.

Delozier's data bring into question the adequacy of a 2-year treatment with TAM as compared to a standard 5-year treatment or longer. When designing our study, we were particularly worried by the carcinogenic effects of TAM, especially in this population of patients with a high likelihood of permanent cure, and decided to limit the treatment duration to 2 years, which had shown an effect in previous randomized studies as demonstrated both in the 1992 and the 1998 Overviews [15,16], with a respective 27% and 24% reduction of recurrences. Subsequent 2005 Overview data [5] continued to indicate a 21% reduction in the risk of recurrence (26% in ER-positive, 11% in ER-poor cases) following 1-2 years of adjuvant TAM. In a recent study [17], 2 years of TAM was able to halve the risk of contralateral breast cancer in premenopausal women of all ages, although the effect was more evident in women younger than 40 years. Interestingly, the protective effect of 2 years of TAM was persistent during the whole follow-up period (median follow-up, 14 years).

Regarding the use of TAM in ER-negative tumours, although the 1998 Overview [16] showed a beneficial effect of TAM, subsequent reports [5,18] indicated a potential deleterious effect of 5 years of TAM. This has not emerged in this study, where TAM was used in a different setting.

At the time this study was designed, the effect of TAM on contralateral breast cancer appeared to be independent of ER status [15,16]. Subsequent studies indicated that its effect is limited to, or prevalent in women who originally had ER-positive breast cancer [5]. In our study, the results thus far are inconclusive on this issue.

## Conclusions

This 5-year analysis has not shown, apart from a smaller number of ER-positive contralateral breast cancers, a statistically significant effect of TAM as used. However, the number of events was low and a longer follow-up with additional events is needed to confirm the trends noted, particularly in terms of reduction in ER-positive contralateral breast cancer, and to possibly add another piece of evidence to the spectrum of activity of this eclectic drug.

Finally, we would like to point out that, although in Western societies the vast majority of patients with ER-positive breast cancer receive postoperative TAM, this is not true in large parts of the world. Strategies of late intervention, therefore, may have a place in the future in that context.

## Competing interests

The authors declare that they have no competing interests.

## Authors' contributions

AV designed the study. AV, GM, MDM, DC, SS and DL managed the patients. EB performed the statistical analysis. All authors participated in the manuscript preparation. All authors read and approved the final manuscript.

## Pre-publication history

The pre-publication history for this paper can be accessed here:

http://www.biomedcentral.com/1471-2407/10/205/prepub
